# Integrins as therapeutic targets in the organ-specific metastasis of human malignant melanoma

**DOI:** 10.1186/s13046-018-0763-x

**Published:** 2018-04-27

**Authors:** Ruixia Huang, Einar K. Rofstad

**Affiliations:** Department of Radiation Biology, Institute for Cancer Research, The Norwegian Radium Hospital, Oslo University Hospital, Ullernchausseen 70, 0379 Oslo, Norway

**Keywords:** Integrin, Organ-specific metastasis, Malignant melanoma, Angiogenesis, Metastasis, Integrin inhibitors, Therapeutic target, Targeted therapies

## Abstract

Integrins are a large family of adhesion molecules that mediate cell-cell and cell-extracellular matrix interactions. Among the 24 integrin isoforms, many have been found to be associated with tumor angiogenesis, tumor cell migration and proliferation, and metastasis. Integrins, especially αvβ3, αvβ5 and α5β1, participate in mediating tumor angiogenesis by interacting with the vascular endothelial growth factor and angiopoietin-Tie signaling pathways. Melanoma patients have a poor prognosis when the primary tumor has generated distant metastases, and the melanoma metastatic site is an independent predictor of the survival of these patients. Different integrins on the melanoma cell surface preferentially direct circulating melanoma cells to different organs and promote the development of metastases at specific organ sites. For instance, melanoma cells expressing integrin β3 tend to metastasize to the lungs, whereas those expressing integrin β1 preferentially generate lymph node metastases. Moreover, tumor cell-derived exosomes which contain different integrins may prepare a pre-metastatic niche in specific organs and promote organ-specific metastases. Because of the important role that integrins play in tumor angiogenesis and metastasis, they have become promising targets for the treatment of advanced cancer. In this paper, we review the integrin isoforms responsible for angiogenesis and organ-specific metastasis in malignant melanoma and the inhibitors that have been considered for the future treatment of metastatic disease.

## Background

Integrins are a large family of heterodimeric transmembrane glycoproteins that mediate cell-cell and cell-cellular environment interactions. They are widely expressed on many types of cells, including tumor cells, endothelial cells (ECs), pericytes, fibroblasts and immune cells [1]. Integrins are composed of two subunits: subunit α, with a size of 120–170 kDa, and subunit β, with a size of 90–100 kDa [2]. In humans, there are 18 α subunits and 8 β subunits that can assemble 24 different integrins with different binding properties, tissue distributions and biological functions [3, 4]. Both α and β subunits possess a large extracellular domain, a transmembrane domain and usually a small cytoplasmic tail [3]. In cancer cells, the extracellular domain serves as a receptor for adhesion proteins and growth factors in the extracellular matrix (ECM) [4], and the cytoplasmic tail is linked to the cytoskeleton and cellular signaling pathways, such as focal adhesion kinase (FAK) and Src family kinases [5], mediating tumor cell attachment, invasion, and migration, as well as tumor angiogenesis [1, 6–8]. Both integrin subunits are required for interactions with the cytoskeleton and ECM [9].

Malignant melanoma is a highly aggressive tumor and has a poor prognosis, especially when it has developed metastases [10]. Early cutaneous melanoma grows horizontally through the epidermis. This is called the radical growth phase (RGP) and is characterized as indolent with a lack of metastatic capability [11]. Over time, the vascular network is enriched, and melanoma cells replicate and increasingly express integrins and other adhesion molecules, which enables the melanoma cells to adhere to the vascular wall and invade the vessels [12]. At this point, melanoma begins the vertical growth phase (VGP), and the tumor begins to thicken and acquires the capability of metastasis. The expression levels of many integrins are changed when the melanoma growth is converted from the RGP to the VGP [13] to enable the tumor cells to interact with their environment, establishing an appropriate vascular network and acquiring a migratory and invasive phenotype. For instance, β1 and β3 integrins are up-regulated in many primary and metastatic melanoma cells in the VGP compared with those in the RGP, and the up-regulation is positively associated with the development of melanoma metastasis [14, 15]. The over-expression of integrin αvβ3 by melanoma cells was also found to play an important role in converting melanoma from the non-tumorigenic RGP to the tumorigenic and invasive VGP [16].

The process of melanoma cell metastasis from a primary tumor to a secondary distant organ usually involves the following steps: detachment from the primary tumor, invasion into blood or lymphatic vessels (intravasation), survival in the circulation, adhesion to blood or lymphatic vessel wall, evasion from the vessels (extravasation), and arrest and colonization in a secondary organ. Integrin-mediated cell adhesion and migration are essential for the whole process of melanoma metastasis development. For example, αvβ3 plays an important role in the transendothelial migration of melanoma cells by binding to adhesion molecule L1 on ECs in the tumor microenvironment (TME) [17]. In addition, in those melanoma cells that do not express β3 integrins, β1 integrins instead play a role in promoting the transendothelial migration of melanoma cells by binding to vascular cell adhesion molecule 1 (VCAM-1) on ECs in the TME [18].

The site of distant metastasis is an important and independent predictor of the survival of melanoma patients with metastatic disease [19, 20]. A recent large-scale cohort study showed that the median overall survival (OS) of melanoma patients with subcutaneous/lymph node metastasis (M1a) was 20.8 months, whereas the melanoma patients with metastasis to the lungs (M1b) had a median OS of 13 months, those with metastasis to the liver/digestive tract (M1c) showed an OS of 5.5 months, those with metastasis to the bone showed an OS of 3.6 months, and those with metastasis to the central nervous system (M1c) showed an OS of only 2.5 months [21]. Because of the large prognostic differences among types of organ-specific metastasis in melanoma, many studies have been focused on organ-specific metastasis with the aim of identifying not only the mediators responsible for directing metastatic melanoma cells to different organs but also therapeutic targets for metastatic melanoma. The distant metastatic sites of melanoma are influenced by communications between the circulating tumor cells (CTCs) and the microenvironment of the targeted organ. One of the most important communications for manipulating organ-specific metastasis is the interaction between adhesion molecules and their receptors integrins. The expression of different integrins by melanoma cells facilitates the arrest and colonization of the melanoma cells in different organs [22]. For instance, melanoma cells expressing integrin β3 tend to metastasize to the lungs [23], whereas melanoma cells expressing integrin β1 preferentially develop lymph node metastases [23–25]. Furthermore, different integrins expressed on the ECs of the targeted organ may also regulate the integrity of its microvasculature, allowing extravasation of CTCs into that specific organ.

## Integrins in angiogenesis and lymphangiogenesis

Tumor cells metastasize to a distant organ by hematogenous and lymphogenous routes. Angiogenesis and lymphangiogenesis, which are mainly regulated by a series of growth factors and receptors, are critical in the above two metastatic routes. Among these growth factors and receptors, vascular endothelial growth factors (VEGFs) and vascular endothelial growth factor receptors (VEGFRs) are especially important to regulate the growth and maintenance of blood and lymphatic vessels. Additionally, angiopoietins (ANGs) and Tie receptors have been regarded in recent decades as the second important axis for regulating the maturation and plasticity of the vessels [26, 27]. Integrins, known as adhesion molecules that modulate cell-cell and cell-matrix interactions, have been identified to be expressed on ECs, lymphatic endothelial cells (LECs) and pericytes [28, 29] and participate in tumor angiogenesis by interacting with both the VEGF-VEGFR [30] and ANG-Tie [31–33] pathways. The roles that different integrin subunits play in angiogenesis have been reviewed elsewhere [28]. Briefly, integrin subunits α1, α2, α3, α4, α5, α6, α9, αv, β1, β3 and β5 are involved in the process of physiological or pathological angiogenesis [28]. The most important subunits, to our current knowledge, that contribute to tumor angiogenesis and lymphangiogenesis will be discussed here.

Among the integrins on ECs, αvβ3 is the most abundant and influential receptor regulating angiogenesis [34–36]. Activated αvβ3 is co-localized with VEGFR-2 on the ECs of proliferating blood vessels [36]. The integrin β3 subunit on ECs, phosphorylated by VEGF-stimulated c-Src, in turn promotes the phosphorylation and activation of VEGFR-2 [30, 35, 36]. The parallel expression of α2bβ3 and αvβ3 integrins by melanoma cells could up-regulate the expression of basic fibroblast growth factor (bFGF) and promote the angiogenic phenotype [37]. Moreover, integrin αvβ3 is required for the survival and maturation of newly formed blood vessels, and αvβ3 antagonists have been shown to induce the apoptosis of proliferative angiogenic ECs [38].

Another important member in the αv family is integrin αvβ5, which is important for neuropilin 1 (NRP-1)-dependent angiogenesis and tumor aggressiveness in melanoma. Evidence has shown that when integrin αvβ5 was inhibited, NRP-1 as a co-receptor of VEGF-A was also blocked, and thus the NRP-1-dependent angiogenesis and aggressiveness of melanoma was also reduced [39].

Integrin β1 is the most abundantly expressed integrin subunit, and it heterodimerizes with at least 12 α subunits, forming 12 different isoforms [40]. Integrin β1 could be directly activated by angiopoietin 2 (ANG-2), consequently leading to endothelial destabilization [41]. Integrin β1 is important for B16 melanoma cells to adhere to ECs both in vivo and in vitro [42]. The activation of integrin β1 in blood cells can also be regulated by “inside-out” signals, leading to metastatic tumor cell extravasation from the circulation into tissues [43]. In melanoma, activated integrin β1 is required for the attachment of metastatic melanoma cells to the vascular basement membrane via regulating the downstream FAK/paxillin pathway [44], and this integrin helps the extravasation of metastatic melanoma cells into the liver [43] and lungs [45]. After the intravenous injection of melanoma cells, liver colonization was found to be significantly increased in animals with melanoma expressing activated integrin β1 compared with that expressing wild-type integrin β1 [43].

The dimerization of subunits β1 and α5, integrin α5β1, is the only known α5 integrin and has been clearly defined as a proangiogenic factor [46, 47]. Integrin α5β1 directly interacts with Tie2 and regulates ANG-1-dependent angiogenesis through this interaction [33]. Additionally, another β1 dimer, α9β1, was found to directly bind to 121 isoforms of VEGF-A, and the blockade of α9β1 specifically inhibited angiogenesis induced by VEGF-A165 and VEGF-A121 [48].

In addition, several integrins, including the previously mentioned αvβ3, αvβ5 and α5β1, serve as receptors for ANG-2 in the absence of Tie2 [32, 49] and induce the enhancement of VEGF-mediated sprouting and FAK (Tyr397) phosphorylation [32, 50].

Less is known about the association between integrins and lymphangiogenesis. Integrin β1, including the α4β1, α2β1, α1β1 and α9β1 isoforms, may participate in the process of tumor-associated lymphangiogenesis [51, 52]. Studies have shown that all or 92% of human melanomas with pathologically positive lymph node involvement expressed integrin β1, whereas only 26% or fewer of melanomas with pathologically negative lymph node involvement showed integrin β1 expression [25, 53]. Integrin α9β1 may contribute to lymphangiogenesis by directly binding to the key lymphangiogenic factors VEGF-C and VEGF-D [52]. Additionally, the α4 integrin subunit may up-regulate VEGF-C expression and promote lymphangiogenesis together with VEGF-C in human colon cancer [54]. Furthermore, in melanoma, α4 expressed on melanoma cells binds to its counter receptor VCAM-1 expressed on LECs with a high affinity and induces the adhesion of melanoma cells to LECs [55], indicating that α4 may participate in lymphangiogenesis and lymphatic metastasis in melanoma.

## Integrins and the metastatic niche

Over 100 years ago, Steven Paget proposed the “seed and soil” hypothesis, stating that metastasis is dependent on the interactions between the “seed” (the cancer cells) and the “soil” (the host microenvironment) [56]. It is now established that cancer cells prepare a metastatic niche (also called a pre-metastatic niche) before leaving the primary tumor [57, 58]. Sowing the “seeds” of metastasis requires the action of tumor-secreted factors and tumor-shed extracellular vesicles that enable the “soil” at distant metastatic sites to encourage the outgrowth of incoming cancer cells [57]. In the pre-metastatic niche, immune cells, ECs and stromal cells, together with growth factors, chemokines, matrix-degrading factors and adhesion molecules, collaborate to accelerate assembly of the metastatic lesion [59]. Compelling evidence has shown that metastatic colonization can only successfully occur in certain organs, although tumor cells reach the vasculature of all organs [60, 61]. Indeed, the organotropic metastasis is rather determined by the specific metastatic niche established at different hosts [57].

Integrins in the metastatic niche participate to regulate immune cell activity and myeloid cell differentiation and function; most importantly, integrins are secreted by tumor cells and transported via exosomes to a distant organ to prepare the metastatic niche. Exosomes are small membranous extracellular vehicles (30–150 nm) that contain functional biomolecules (including proteins, lipids and nucleic acids) [62]. Tumor-derived exosomes bud off from tumor cells and transport different biomolecules to distant cells in the body, inducing vascular leakage, inflammation and bone marrow progenitor cell recruitment during pre-metastatic niche formation [63]. Indeed, tumor-derived exosomes play a vital role in developing organ-specific metastasis [57]. Different integrins on the surface of exosomes were recently found to play important roles in preparing favorable pre-metastatic niches in specific organs [64–66]. For instance, exosomal integrins α6β4 and α6β1 preferentially direct circulating melanoma cells to the lungs, whereas exosomal integrin αvβ5 induces liver metastasis [65]. Targeting integrins α6β4 and αvβ5 resulted in decreased lung and liver metastasis, respectively [65].

## Integrins and organ-specific metastasis in malignant melanoma

As discussed above, integrins and their downstream signaling are vastly involved in regulating the vasculature, angiogenesis, the immune response and the stromal context of the metastatic niche, but different integrins are involved in inducing organ-specific metastasis. Different integrins that contribute to the organ-specific metastasis of malignant melanoma and promising inhibitors are summarized in Table 1.

**Table 1:** The responsible integrins for the organ-specific metastasis of human melanoma and the inhibitors of these integrins

Integrin	Metastatic site	Inhibitor
α4	Lymph node [55]	TBC3486 [141] JK273 [144] Natalizumab (Antegren) [139, 140]
α4β1	Lymph node [29, 53, 55, 138], bone [98]	TBC3486 [141] JK273 [144]
β1	Lymph node [18, 53, 138]	AIIB2 [145]
α2	Liver [92]	–
α2β1	Lung [82], liver [82]	BTT-3033, BTT-3034 [146]
α5β1	Liver [96]	Volociximab (M200) [128] ATN-161 [95, 133–135] PF-04605412 [137]
β3	Bone [101]	Abergrin (eteracizumab, MEDI-522) [121, 122] MK-0429 [76]
αv	Brain [110]	Intetumumab (CNTO 95) [113] Cilengitide (EMD 121974) [120]
αvβ3	Lung [76], bone [97], brain [108, 111],	MK-0429 [76] Abergrin (eteracizumab, MEDI-522) [121, 122] Cilengitide (EMD 121974) [120]

### Integrins and lymph node metastasis

Regional lymph node metastasis is an early sign of malignant spread and associated with a poor prognosis in melanoma patients. The development of lymph node metastasis starts with the following two steps: (1) the growth of lymphatic vessels (lymphangiogenesis) at the tumor periphery; and (2) the recruitment of melanoma cells into lymphatic vessels, which is defined as lymphatic invasion [67–69]. Some integrins, including α4β1, α2β1, α1β1 and α9β1, participate in the process of lymphangiogenesis at the tumor periphery [51, 52], and some integrins, such as α9β1 and α4, help with the recruitment of melanoma cells into lymphatic vessels [52, 55].

**Fig. 1:**
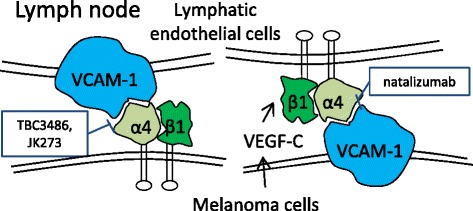
Integrin that is responsible for lymph node metastasis in melanoma and its inhibitors. Integrin α4, especially α4β1 isoform, promotes the adhesion of melanoma cells to the lymphatic endothelial cells (LECs) via binding to VCAM-1. α4β1^+^ melanoma cells tend to adhere to VCAM-1^+^ LECs, and α4β1^+^ LECs have high affinity to VCAM-1^+^ melanoma cells as well. The lymphangiogenic growth factor VEGF-C, which is secreted by tumor cells and transported to ECM, is supposed to induce the expression and activation of integrin α4β1 on LECs. Monoclonal antibody natalizumab and small molecules TBC3486 and JK273 are inhibitors of integrin α4β1

Among those integrins, integrin α4 is especially important in the processes leading to the lymph node metastasis of melanoma. It has been reported to be associated with tumorigenicity and lymph node metastasis in many malignancies, including colon cancer [54], lung cancer [70], pancreatic ductal carcinoma [70] and melanoma [55]. Integrin α4 can dimerize with β1 and β7 subunits, forming two isoforms, α4β1 (VLA-4) and α4β7, in which α4β1 is especially important for the adhesion of melanoma cells to LECs via binding to VCAM-1 (Fig. 1) [18, 55]. On the one hand, α4β1 is expressed on some melanoma cells and helps them to attach to the VCAM-1^+^ LECs [18, 55]. On the other hand, integrin α4β1 is also expressed on LECs in the lymph nodes, and activated α4β1 on LECs in lymph nodes plays a functional role in capturing VCAM-1^+^ metastatic melanoma cells [29]. The lymphangiogenic growth factor VEGF-C, which is secreted by tumor cells and transported to the ECM, is supposed to induce the expression and activation of integrin α4β1 on LECs [29, 70]. The suppression of integrin α4β1 in LECs significantly prevents lymphangiogenesis at the tumor periphery and lymph node metastasis [70]. Taken together, antagonists of integrin α4β1 are promising for inhibiting the interactions of melanoma cells with the lymph node microenvironment and suppressing lymph node metastasis.

### Integrins and lung metastasis

**Fig. 2:**
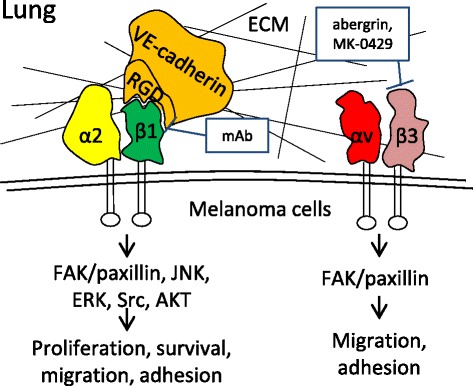
Integrins that are responsible for the lung metastasis of melanoma and the inhibitors. VE-cadherin activates α2β1 integrin and the downstream signaling pathway by binding to the β1 subunit with the RGD motifs, and the activation of the α2β1 integrin pathway promotes tumor cell invasion and transendothelial migration, thus inducing lung and liver metastases. Blocking the interactions between integrin α2β1 and cadherin RGD motifs with highly selective monoclonal antibodies (mAb) significantly reduced the incidence of lung metastasis and improved the survival rate of the experimental mice. Integrins αvβ3, as a proangiogenic factor, plays an important role in directing circulating melanoma cells to the lungs and eventually leading to pulmonary metastasis by enhancing the tumor cell adherence to the pulmonary vasculature. Integrins αvβ3 inhibitors abergrin and MK-0429 selectively bind to β3 subunit and reduce the incidence of pulmonary metastasis in melanoma mouse model

The lungs are the most commonly involved when melanoma metastasizes to a distant organ [21, 71]. Our previous study showed that lung metastasis in melanoma was mainly generated by the hematogenous route and was associated with angiogenic activity and pro-angiogenic genes [72]. Based on this aspect, the arrest of melanoma cells in the pulmonary microvasculature and the colonization of melanoma cells in the lungs are required for developing lung metastasis. Several integrins, such as αvβ3 and α2b, play important roles in helping circulating melanoma cells adhere to the vascular wall in the pulmonary microenvironment [73, 74], and some other integrins, such as β1, intensify the colonization capability of metastatic melanoma cells in the lungs (Fig. 2) [75].

Integrin αvβ3, a receptor for the ECM proteins vitronectin and fibronectin, is well characterized as a proangiogenic factor in melanoma. The expression of αvβ3 on breast cancer cells specifically directs tumor cells to the lungs and bones and promotes spontaneous metastasis to the lungs and bones from the mammary glands in a preclinical in vivo model [73]. Nevertheless, αvβ3 does not promote the proliferation of breast cancer cells in vitro or in the primary site in vivo, suggesting that αvβ3 may participate in enhancing tumor cell adherence to the pulmonary vasculature but not in promoting the proliferation of tumor cells [73]. Likewise, integrin αvβ3 in melanoma is required for the successful establishment of a pulmonary metastasis model by the tail vein injection of B16-F10 melanoma cells [76]. Treatment with the αvβ3 inhibitor MK-0429 after the tail vein injection significantly reduced the rate of metastasis in the lungs compared with the vehicle treatment, showing the important role of αvβ3 in specifically directing circulating melanoma cells to the lungs, eventually leading to pulmonary metastasis [76].

Integrin αvβ5, another proangiogenic factor, is considered to induce carcinoma invasion and metastasis but not influence the proliferation of tumor cells in the primary site or in vitro [77]. It exerts this function at least partly by interacting with epidermal growth factor (EGF) and EGF receptor (EGFR) [77–79]. Integrin αvβ5, especially the β5 subunit, was found to be important for the development of pulmonary vascular permeability, mainly by regulating VEGF, transforming growth factor-β (TGF-β) and thrombin [80]. Cilengitide, as an inhibitor of αvβ3 and αvβ5 integrins, was found to inhibit both the primary tumor growth and pulmonary metastasis of osteosarcoma [81]. However, no data have shown its impact on the pulmonary metastasis of melanoma.

GPIIb is the protein produced by the integrin α2b (ITGA2B) gene. It plays a complex role in the development of lung metastasis in melanoma. Highly metastatic B16-D5 melanoma cells were injected intravenously into GPIIb-deficient (GPIIb^−/−^) or WT mice, and tumor cells rapidly accumulated in the pulmonary vasculature in the WT mice but not the GPIIb^−/−^ mice, indicating that the presence of GPIIb in the pulmonary host is required for the arrest of melanoma cells in the lungs to develop lung metastasis [74]. However, surprisingly, the occurrence of lung metastasis was higher in the GPIIb^−/−^ mice than the WT mice, indicating that the absence of integrin subunit α2b may accelerate the colonization of metastatic melanoma cells in the lungs [74]. Therefore, the role of integrin subunit α2b in the development of lung metastasis in melanoma is still controversial.

Integrin β1 and the downstream FAK signaling are considered important for the proliferation of metastatic cancer cells after they extravasate into the lungs [75]. Vascular endothelial-cadherin (VE-cadherin) is expressed in highly aggressive melanoma. VE-cadherin activates the α2β1 integrin pathway by binding to the β1 subunit with arginine-glycine-aspartate (RGD) motifs, and activation of the α2β1 integrin pathway promotes invasion and transendothelial migration, thus inducing lung and liver metastases in vivo [82]. A highly selective monoclonal antibody (mAb) specifically blocked the cadherin RGD-induced activation of α2β1, significantly reduced the incidence of lung metastasis in melanoma and improved the survival rate of the experimental mice [83]. Consequently, integrin β1, especially isoform α2β1, may contribute to the later phase of pulmonary metastasis in melanoma and merits further investigations as a promising target for the treatment of metastatic melanoma.

A lung-specific EC adhesion molecule (Lu-ECAM-1, CLCA2) localized on endothelia of distinct branches of pulmonary blood vessels was identified in the 1990s as a factor that mediates the specific adherence of B16-F10 cells to the lungs [84]. The antibody blocking Lu-ECAM-1 reduced 90% of the lung colonies caused by B16-F10 cell injection [84]. In breast cancer, Lu-ECAM-1 (CLCA2) was capable of facilitating lung metastasis by interactions with integrin α6β4 expressed on breast cancer cells [85]. However, it is not known how Lu-ECAM-1 facilitated the formation of B16-F10 tumor cell colonies in the lungs. To the best of our knowledge, no data about the potential role of α6β4 on melanoma cells in leading to lung metastasis have been reported.

### Integrins and liver metastasis

The liver is another common organ for melanoma metastasis, in addition to the lymph nodes and lungs, because the liver receives a dual blood supply from the portal vein and hepatic arteries, and melanoma is a hypervascular malignancy [86]. From a molecular perspective, this finding is additionally attributed to specific adhesive molecules, such as integrins, that direct metastatic melanoma cells to the liver. Many integrins, especially integrin β1, play important roles in mediating the attachment of hepatocytes to the liver ECM and helping the proliferation and migration of myofibroblasts, leading to liver fibrosis [87–89]. In addition, liver fibrosis is closely linked to the preparation of the premalignant environment in the liver [90, 91].

**Fig. 3:**
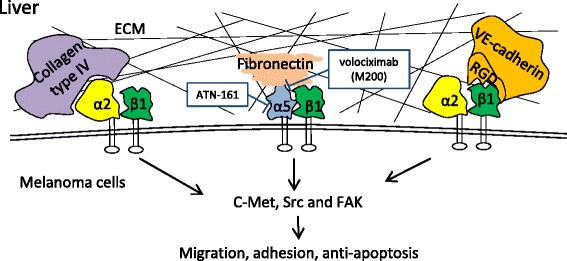
Integrins responsible for the liver metastasis of melanoma and the inhibitors. Integrin α2 and α5 may be involved in mediating liver metastasis in melanoma. Integrin α2 exerts this function mainly via binding to collagen type IV, which is highly present in the liver sinusoids and important for the collagen type IV-dependent activation of FAK. One of the α2 isoforms, α2β1, which is activated by VE-cadherin, was also found to promote liver metastasis of melanoma in a preclinical model. β1 integrins, including α2β1 and α5β1, and the downstream c-Met, Src and FAK pathway may also contribute to liver metastasis by promoting tumor cell invasion and transendothelial migration. Volociximab (M200) and ATN-161 are supposed to inhibit liver metastasis by binding to the α5 subunit of integrin α5β1

Integrin α2 (CD49b) is an important integrin that may contribute to the liver metastasis of melanoma. B16-KY8 is a cell line with a high propensity for forming hepatic nodules; the line was derived from B16-F0 melanoma cells by eight passages in a hepatic metastasis model. Out of a broad array of cell membrane molecules, integrin α2 was uniquely up-regulated in the liver-metastasizing subline B16-KY8 versus the original line B16-F0 [92]. Cells with high and low integrin α2 expression were selected from the B16-KY8 subline by cell sorting, and the cells with high α2 expression caused significantly more hepatic nodules than those with low α2 expression [92]. The selective role of integrin α2 in liver metastasis was further demonstrated by functional studies in which integrin α2 was inhibited and over-expressed in B16 melanoma cells [92]. Similarly, blocking integrin α2 with anti-integrin α2 antibody significantly prevented operation-induced liver metastasis in a rat colon cancer model [93]. Interestingly, blocking B16-KY8 melanoma cells with anti-integrin α2 antibody reduced the number of liver metastases but increased the occurrence of peritoneal growth, indicating that integrin α2 was rather specific for the development of liver metastasis in melanoma [92]. Integrin α2 mediates liver metastasis mainly by binding to collagen type IV, which is highly present in the liver sinusoids and important for the collagen type IV-dependent activation of FAK [92]. One of the integrin α2 isoforms, α2β1, which is activated by VE-cadherin, was found to promote liver metastasis in preclinical melanoma and breast cancer models (Fig. 3) [82].

Integrin α5β1 is the only known α5 integrin and seems to be an upstream factor of c-Met, Src and FAK [94]. The inhibition of integrin α5β1 has reduced the liver metastasis rate of ovarian cancer [94] and colorectal cancer [95] in mouse models. In a very recent study, integrin α5 was reduced when the liver metastasis of melanoma was suppressed by treatment with the mitogen-activated protein (MEK) inhibitor selumetinib [96], suggesting the promise of integrin α5 as a therapeutic target for liver metastasis in melanoma. However, limited investigations on melanoma have been performed to date.

### Integrins and bone metastasis

Integrins on both melanoma cells and host stromal cells (osteoclasts, vascular cells, inflammatory cells, platelets and bone marrow stromal cells) in bone play important roles in promoting bone metastasis [97].

**Fig. 4:**
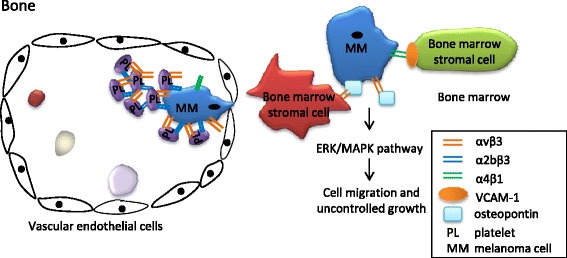
Integrins responsible for the bone metastasis of melanoma. Integrins αIIbβ3 and αvβ3 play important roles in the adhesion of melanoma cells to platelets and platelet aggregation, which are important for the adherence of circulating melanoma cells to the capillaries of bone. The over-expression of integrin α4β1 on primary melanoma cells was found to be associated with increased bone metastasis, probably via the interaction with VCAM-1, which is constitutively expressed on bone marrow stromal cells. Integrin αvβ3 on melanoma cells is activated by binding to its ligand osteopontin, which is abundant in bone matrix. The activated integrin αvβ3 may enhance the ability of cell migration and thus induce bone metastasis probably via the downstream ERK/MAPK pathway

Integrin signaling through α4β1 and αvβ3 on tumor cells may promote tumor cell metastasis to and proliferation in the bone microenvironment (Fig. 4) [97]. The over-expression of integrin α4β1 on primary melanoma cells was found to be associated with increased bone metastasis, probably via interaction with VCAM-1, which is constitutively expressed on bone marrow stromal cells [98]. Integrin αvβ3 plays an important role in generating new blood vessels, which is needed for tumor growth [38]. In addition, the αvβ3 ligand osteopontin on melanoma cells also promotes melanoma bone metastasis, probably through the ERK/MAPK pathway [99]. Osteopontin is an RGD-containing protein and promotes the attachment of melanoma cells to the bone microenvironment, where it is abundant [100]. Osteopontin in the bone marrow is needed for the growth of B16 melanoma cells implanted in the bone [100].

B16 melanoma cells were injected into the left cardiac ventricle of β3^+/+^ and β3^−/−^ mice, and 74% of the β3^+/+^ mice developed osteolytic bone metastasis by 14 days, whereas only 4% of the β3^−/−^ mice developed bone lesions [101], confirming the role of host cell β3 integrin expression during the process of melanoma bone metastasis. A functional study also showed that integrin β3 in the bone marrow was required for the functionality of newly formed blood vessels [102]. The β3 integrin subunit heterodimerizes with two α subunits, forming isoforms α2bβ3 (GPIIbIIIa) and αvβ3 [103]. Both isoforms are required for the adhesion of melanoma cells to platelets [104] and platelet aggregation [97, 105], which are important for the capture of melanoma cells in the capillaries of bone.

### Integrins and brain metastasis

**Fig. 5:**
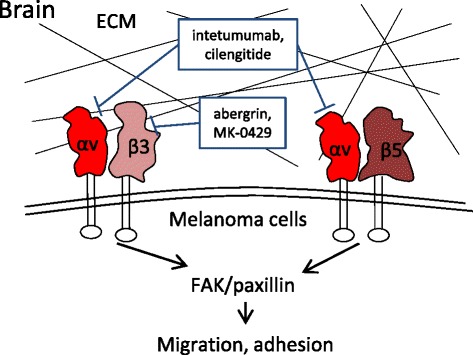
Integrins responsible for the brain metastasis of melanoma and the inhibitors. Integrin αv, including αvβ3 and αvβ5, promotes the cell migration rate and the adherence of circulating melanoma cells to the brain vasculature, consequently increasing the occurrence of brain metastasis. Abergrin and MK-0429 selectively inhibit integrin αvβ3 by binding to the β3 subunit, whereas intetumumab and cilengitide inhibit both integrins by binding the αv subunit

Due to the presence of the blood-brain barrier, the treatment of brain metastasis with recently developed targeted therapies and immunotherapies is not as effective as that of extracerebral metastases in melanoma. Thus, it remains a major challenge for the treatment of patients with malignant melanoma, and studies on brain-specific targeted therapeutics are warranted. The expression of αv integrins was significantly up-regulated in the brain metastases of several solid tumors, including melanoma, compared with the corresponding primary tumors [106–109], indicating the role of αv integrins in helping tumor cells penetrate the blood-brain barrier and colonize in the brain parenchyma (Fig. 5). The expression of several αv integrins, such as αvβ3, αvβ5 and αvβ8, on melanoma cells is up-regulated in the brain metastases compared with that in the primary tumor [108]. The over-expression of integrin αv in melanoma cells was found to accelerate the cell migration rate in vitro and promote melanoma cells to adhere to the brain vasculature in vivo, consequently increasing the occurrence of brain metastasis in an athymic rat model [110]. These findings suggest that integrin αv is promising as a therapeutic target for the brain-specific metastasis of melanoma.

Among these αv integrins, αvβ3 may play a specific role in inducing the brain metastasis of melanoma. The melanoma cell lines Mel57 and Zkr, which express αvβ3, yielded metastasis reproducibly in the brain parenchyma, while other melanoma cell lines, MV3 and BLM, which do not express αvβ3, only preferentially metastasized to the dura mater and leptomeninges instead of the parenchyma [111]. Nevertheless, a functional study by αvβ3 transfection in the BLM melanoma cell line did not show any differences in the metastatic pattern [111].

## Integrins as targets for melanoma therapies

Therapeutic drugs that target integrins mainly include three forms: therapeutic antibody-based drugs, peptide-based drugs and small molecule-based drugs; each form has its own advantages and limitations [112]. Antibody-based drugs have high target specificity and affinity and thus less toxicity. Additionally, they can be modified to be more specific and less toxic, but the high cost of production and the need for intravenous administration limit their production and clinical use. Peptide-based drugs are easier to design as most integrin ligands and their recognition consequences have been determined. They usually have moderate to high affinity but may lack specificity since the same ligand can be shared by many integrins. They also have additional disadvantages, such as the need for injection, a high production cost and limited stability. In addition to being readily synthesized, less costly, and more stable, small molecule-based drugs can be administered orally. Most of these drugs require high-throughput library screening. These small molecule antagonists generally have limitations in terms of bioavailability, serum protein binding and integrin selectivity. Many therapeutic drugs for melanoma have been under investigation in both preclinical studies and clinical trials (Table 2).

**Table 2:** Integrin inhibitors and their effect on metastatic melanoma in preclinical studies and clinical trials

Integrin Inhibitor	Targeted integrin	Effect on metastatic melanoma	Clinical trial	Phase of the clinical trial
MK-0429	αvβ3	Reduce lung metastasis of melanoma in mouse models [76]	–	–
Intetumumab (CNTO 95)	αv	Inhibits melanoma cell adhesion, migration and invasion in vitro. Inhibit tumor growth in mouse xenografts of melanoma.	NCT00246012	Phase I/II [116]
Abergrin (eteracizumab, MEDI-522)	αvβ3	Patients treated with Abergrin + decarbacine did not show survival benefit compared to decarbacine alone.	NCT00111696 NCT00066196	Phase I [147], Phase II [122]
Cilengitide (EMD 121974)	αvβ3 and αvβ5	It was well tolerated but achieved minimal efficacy when used as a single-agent treatment.	NCT00082875	Phase II [120]
Volociximab (M200)	α5β1	It was well tolerated at 10 mg/kg Q2W, and achieved preliminary clinical effect: SD was observed in 32/37 (87%) of patients.	NCT00099970	Phase II [130]

*Note*: *Q2W* every 2 weeks, *SD* stable disease

### Inhibitors of *α*v integrins

As discussed elsewhere in this paper, αv integrins, especially αvβ3 and αvβ5, play an important role in tumor angiogenesis by interacting with the VEGF-VEGFR and ANG-Tie systems. A fully human anti-αv integrin mAb, intetumumab (CNTO 95), was developed, and it has been shown to prevent angiogenesis and tumorigenesis in human melanoma xenografts in both nude mice and nude rats [113]. Interestingly, the effect of intetumumab on inhibiting tumor growth and tumor metastasis is more likely not dependent on its anti-angiogenic activity because this antibody only recognized αvβ3 and αvβ5 on human melanoma cells, not mouse angiogenic integrins [113]. Furthermore, intetumumab increased the sensitivity of radioresistant tumor cells, including M21 melanoma cells, to fractionated radiotherapy in an in vivo model [114]. Due to the promising results of preclinical studies, clinical studies have been designed to examine the efficacy of intetumumab for treating human metastatic melanoma. To date, it has been enrolled in phase I [115] and phase II [116] clinical trials for treating melanoma and showed tolerable toxicity. Patients with stage IV melanoma were treated with dacarbazine and 10 mg/kg intetumumab compared with dacarbazine and a placebo. In terms of the clinical endpoint, no significant benefit was achieved from the regimen with intetumumab [116], possibly due to the limited number of patients enrolled; yet, health-related quality of life seemed to be improved in the patients treated with dacarbazine and intetumumab compared with those treated with dacarbazine and a placebo [117]. Larger-scaled studies on the promising efficacy of intetumumab in the treatment of melanoma and prostate cancer are warranted, but the development of the drug was discontinued by the original company, Centocor, Inc. [118].

Cilengitide (EMD 121974) is another inhibitor of integrins αvβ3 and αvβ5. It has shown an anti-angiogenic effect and a promising antitumor effect in many cancers by inhibiting the binding of integrins αvβ3 and αvβ5 to the ECM [81, 119]. A randomized phase II clinical trial has been completed to evaluate the antitumor effect of cilengitide in patients with metastatic melanoma. The results showed that the drug was well tolerated but achieved minimal efficacy when used as a single-agent treatment [120]. Interestingly, the sole responder and one of two patients with stable disease had no αvβ3 expression at baseline, indicating that its clinical efficacy was independent of αvβ3 expression at baseline [120]. Likewise, in vitro studies found that cilengitide markedly decreased the invasiveness and angiogenic activity of melanoma cells by the inhibition of αvβ5 instead of αvβ3 [39]. To conclude, existing studies have shown that cilengitide exerts anti-angiogenic and anti-metastatic functions in an integrin αvβ5-dependent and integrin αvβ3-independent manner.

However, in addition to integrin αvβ5, integrin αvβ3 is also important for tumor angiogenesis and tumorigenesis. Integrin αvβ3 is required for the survival and maturation of newly formed blood vessels, and an αvβ3 antagonist has been shown to induce the apoptosis of proliferative angiogenic ECs [38]. Several inhibitors that selectively target αvβ3 have been produced and have shown promising antitumor results in metastatic melanoma.

MK-0429 is a selective αvβ3 inhibitor, which was synthesized by Merck & Co., Inc. It was primarily used in prostate cancer and metastatic bone disease but was discontinued due to insufficient clinical benefits. Data from this company later reported promising results for the treatment of metastatic melanoma in preclinical studies, providing evidence that MK-0429 significantly reduced the lung metastasis of melanoma in a mouse model [76]. However, no clinical trials have been performed to date.

Another αvβ3 inhibitor, abergrin (etaracizumab, MEDI-522), manufactured by MedImmune, Inc., is a humanized mAb being investigated for the treatment of metastatic melanoma, prostate cancer, ovarian cancer and various other types of cancer. It has been used in metastatic melanoma in phase I [121] and phase II [122] clinical trials, showing tolerable side effects but unsatisfactory efficacy. Likewise, in metastatic melanoma, treatment with abergrin + dacarbazine did not achieve a relevant survival benefit compared with dacarbazine alone [123].

LM609 is a mouse mAb that specifically recognizes human integrin αvβ3 and inhibits angiogenesis [124]. It showed promising results for inhibiting angiogenesis, the tumor cell invasion ability and tumor growth in breast cancer and melanoma in the 1990s [38, 125]. Later, it was verified that the treatment of melanoma cell lines with LM609 or αvβ3 siRNA yielded similar results. Both strategies down-regulated the expression of αvβ3 and its downstream molecules FAK and PAK1, induced tumor cell apoptosis [126], and dramatically decreased the ability of tumor cell adhesion and invasion [127]. No in vivo studies or clinical trials have been performed.

### Integrin α5β1 inhibitors

As the only known α5 integrin, α5β1 has been defined as a proangiogenic factor and may be associated with liver metastasis in melanoma [96]. Volociximab (M200) is a chimeric mAb that specifically binds integrin α5β1 and blocks the interaction between integrin α5β1 and its ligand, fibronectin [128]. It has shown promising activity in different types of cancer in preclinical and clinical studies, which has been reviewed elsewhere [128]. A phase I clinical trial using escalating doses of volociximab has shown that it can be administered at up to 15 mg/kg per week for solid tumors [129]. In the same year, a multicenter phase II study of volociximab in patients with relapsed metastatic melanoma revealed that weekly volociximab treatment at 15 mg/kg was well tolerated and achieved preliminary clinical efficacy [130]. More clinical studies using volociximab as a single-drug treatment or combined with chemotherapy to treat other metastatic solid tumors have been performed since then to better understand its pharmacokinetics and clinical efficacy [131, 132].

ATN-161 is a small peptide that interacts with the N-terminus of the β1-domain of integrin α5β1 and inactivates integrin α5β1 [95]. ATN-161 inhibited VEGF-induced cell migration and capillary tube formation in ECs [133], suggesting that it has a potent anti-angiogenesis effect on tumors. Preclinical and clinical studies have shown that treatment with ATN-161 alone or together with chemotherapy not only reduces tumor angiogenesis and liver metastasis but also improves patient survival [95, 134, 135]. Intriguingly, it seems that ATN-161 more likely interacts with integrin α5β1 on activated ECs instead of integrin α5β1 on tumor cells because ATN-161 reduced the in vivo growth of xenograft human colon cancer cells (HT29) not expressing α5β1 [136].

A new α5β1 inhibitor, PF-04605412, which is a fully human, Fc-engineered IgG1 mAb, has failed in a phase I clinical trial on human patients with solid tumors because no antitumor effect was observed [137]. This finding may be due to the limited number of patients and the different solid tumors with variable stages included. In this case, modified regimens for heterogeneous patients with different tumors may be helpful.

### Inhibitors of *α*4 integrins

There are two members in the α4 integrin family, α4β1 and α4β7. Integrin α4β1 is specifically responsible for lymph node metastasis in melanoma [29, 53, 55, 138]. Natalizumab (Antegren, Tysabri) is a humanized mAb, which selectively blocks *α*4 integrins on the surface of lymphocytes, thereby preventing their adhesion to VCAM-1. It was approved internationally for the treatment of multiple sclerosis (MS). However, natalizumab seems to be associated with the development of melanoma in MS patients [139, 140]. The FDA’s Adverse Event Reporting System (FAERS) (2004–2014) includes 137 natalizumab-associated melanoma reports in MS patients [140]. Among those patients, 34% were diagnosed with melanoma within 2 years of natalizumab treatment [140]. Consequently, natalizumab, which may lead to the occurrence of melanoma, is unlikely to be used as a therapeutic agent for metastatic melanoma.

However, natalizumab inhibits both α4β1 and α4β7, which may make its effect slightly more complicated [141]. In contrast, TBC3486 is a small molecule that is 200-fold more potent in inhibiting α4β1 than α4β7 [141]. In addition, it is completely inactive against all other integrins tested, including β1, β2 and β3 integrin family members [141]. Hsieh YT and colleagues have shown in recent years that TBC3486 can sensitize drug-resistant acute lymphoblastic leukemia to chemotherapy [141, 142]. However, to date, no preclinical or clinical studies on the effect of TBC3486 on melanoma have been published.

JK273 is a small molecule inhibitor of integrin α4. It was identified by Lee J. and colleagues through a cell-based screen of small molecule libraries and has been shown to inhibit integrin α4-dependent cell migration [143]. Recently, the same group reported that this small molecule exerted a selective cytotoxic effect against non-small cell lung cancer NCI-H460 cells [144]. Further studies are warranted to determine its antitumor effect on other solid tumors, including melanoma.

## Conclusions

Integrins, especially αvβ3, αvβ5 and α5β1, participate in mediating tumor angiogenesis by interacting with the VEGF and angiopoietin-Tie signaling pathways. Integrin subunits that show a clear association with the organ-specific metastasis of human malignant melanoma include α4 and β1 for lymph node metastasis, β3 for lung and bone metastasis, α2 for liver metastasis and αv for brain metastasis. Although many different drugs targeting a variety of integrins have been developed, none of them have shown sufficient evidence for their clinical use in patients with metastatic melanoma. To conclude, the use of a single integrin as a therapeutic target for metastatic melanoma is not a promising approach because different integrins are responsible for angiogenesis and organ-specific metastasis in human malignant melanoma. However, toxicity is a challenging problem if several integrins are targeted simultaneously due to their prevalence and extensive involvement in maintaining normal biological and physiological functions.

## Abbreviations

ANG-2: Angiopoietin 2
bFGF: Basic fibroblast growth factor
CTCs: Circulating tumor cells
ECM: Extracellular matrix
ECs: Endothelial cells
EGF: Epidermal growth factor
EGFR: Epidermal growth factor receptor
FAERS: FDA’s Adverse Event Reporting System
FAK: Focal adhesion kinase
LECs: Lymphatic endothelial cells
Lu-ECAM-1: Lung-specific EC adhesion molecule
mAb: Monoclonal antibody
MEK: Mitogen-activated protein
NRP-1: Neuropilin 1
OS: Overall survival
RGD motifs: Arginine-glycine-aspartate motifs
RGP: Radical growth phase
TGF-β: Transforming growth factor-β
TME: Tumor microenvironment
VCAM-1: Vascular cell adhesion molecule 1
VE-cadherin: Vascular endothelial-cadherin
VEGFRs: Vascular endothelial growth factor receptors
VEGFs: Vascular endothelial growth factors
VGP: Vertical growth phase
